# An Architecture for Computer-Aided Detection and Radiologic Measurement of Lung Nodules in Clinical Trials

**Published:** 2007-05-12

**Authors:** Matthew S Brown, Richard Pais, Peiyuan Qing, Sumit Shah, Michael F McNitt-Gray, Jonathan G Goldin, Iva Petkovska, Lien Tran, Denise R Aberle

**Affiliations:** 1 Department of Radiological Sciences, David Geffen School of Medicine at UCLA, Los Angeles, CA, U.S.A

**Keywords:** “Computer-Aided Diagnosis”, “Lung Nodules”, “CT”

## Abstract

Computer tomography (CT) imaging plays an important role in cancer detection and quantitative assessment in clinical trials. High-resolution imaging studies on large cohorts of patients generate vast data sets, which are infeasible to analyze through manual interpretation.

In this article we describe a comprehensive architecture for computer-aided detection (CAD) and surveillance on lung nodules in CT images. Central to this architecture are the analytic components: an automated nodule detection system, nodule tracking capabilities and volume measurement, which are integrated within a data management system that includes mechanisms for receiving and archiving images, a database for storing quantitative nodule measurements and visualization, and reporting tools.

We describe two studies to evaluate CAD technology within this architecture, and the potential application in large clinical trials. The first study involves performance assessment of an automated nodule detection system and its ability to increase radiologist sensitivity when used to provide a second opinion. The second study investigates nodule volume measurements on CT made using a semi-automated technique and shows that volumetric analysis yields significantly different tumor response classifications than a 2D diameter approach. These studies demonstrate the potential of automated CAD tools to assist in quantitative image analysis for clinical trials.

## 1. Introduction

Computer tomography (CT) imaging plays an important role in cancer detection, diagnosis and in quantitative assessment in clinical trials. CT data sets contain a large number of images per patient; a typical thin-section thoracic CT scan contains approximately 400 cross-sectional images with 1 mm spacing. A clinical trial usually involves large cohorts of patients, each patient potentially undergoing multiple scans over the trial’s course. Faced with this data overload, computer-assisted tools are being developed to detect and quantitate nodules in CT, and show great potential as an aid in image interpretation.

There has been substantial research into the development of computer-aided detection (CAD) systems that can locate lung nodules and measure their size (1, 2, 3, 4, 5). However, there has been less emphasis on the infrastructure required to integrate these into a clinical trials setting, and enable data mining on derived image measures in large cohorts of patients. CAD systems are being developed, and their impact on reader performance evaluated, though not routinely used in clinical practice or large clinical trials, partly because they are not integrated within a data management infrastructure needed for a trial. Commercially available Picture Archiving and Communication Systems (PACS) focus on storage of image data and are not well suited for efficient storage and mining of CAD or quantitative data.

In this article we describe a comprehensive architecture for CAD and surveillance on lung nodules in CT images. Central to this architecture are the analytic components: an automated nodule detection system, nodule tracking capabilities and volume measurement, and their integration within a data management system that includes mechanisms for receiving and archiving images, a database for storing quantitative nodule measurements and visualization, and reporting tools.

We will describe two studies to evaluate CAD technology within this architecture, and the potential application in large clinical trials. The first study involves performance assessment of an automated nodule detection system and its impact on a radiologist’s sensitivity (6). The second study investigates nodule volume measurements on CT (7). These studies show the potential of CAD tools to assist in quantitative image analysis for clinical trials.

## 2. Methods

### 2.1. Data model

We have developed a generic data and process model for quantitative image analysis within a centralized radiology core facility (8). Here we outline the model and focus on the aspects relevant to lung cancer imaging and assessment. The data model supports image data being collected within multi-center clinical trials and also allows multiple clinical trials to be stored and organized within a single database. This capability offers the potential for data mining across trials, effectively pooling cohorts of subjects.

**Figure 1:**
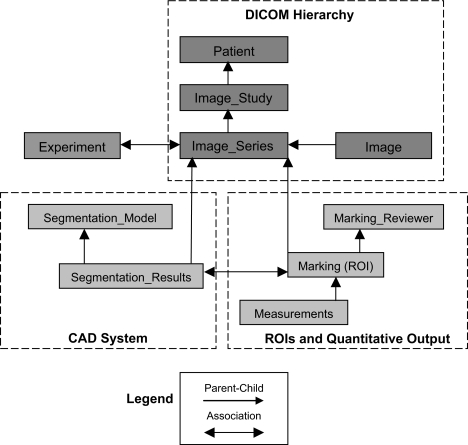
Overview of data model for image, CAD and quantitative data in a clinical trials setting.

Images are received from participating sites in Digital Imaging and Communications in Medicine (DICOM) format (9), transferred in encrypted form (10) to a secure DICOM receiver, and imported into our data server. During the importation, “header information,” including subject identifiers and CT image acquisition parameters (slice thickness, tube current, etc.), is extracted from the images. These fields are represented in the data model using the DICOM hierarchy of patient, imaging study, imaging series and image (Figure 1). Prior to transfer, patient name and other identifiers are replaced in the imager header with a study identifier, thus anonymizing the patient within the database. The image data is too large to be stored directly in the database (approximately 50 MB for a single CT series of 100 images), so the database stores a path to the image files on the server.

Following import, image data can be analyzed using the Quantitative Imaging Workstation (QIWS) developed at UCLA. This workstation has a variety of automated and semi-automated CAD and measurement tools, described in Section 2.2. The quantitative output from these tools includes nodule image coordinates and boundaries, volumes, and attenuation measures. As shown in Figure 1, the data model includes tables storing this quantitative information linked to the image series table. Quantitative measurements are therefore associated with the corresponding CT scan, and can be related back to the subject through the DICOM hierarchy.

To support multiple clinical trials within the architecture, an Experiment table is included within the data model; an entry within the table corresponds to a clinical trial. As shown in Figure 1, a row in the Image_Series can be associated with a row in the Experiment table, allowing subjects and quantitative measurements to be linked with one or more clinical trials. This is necessary to perform database queries relating to a specific trial and also for constructing patient worklists in QIWS for users working on multiple trials.

The data model has been implemented in PostgreSQL: an open-source database (11). This currently includes images and derived quantitative data, but could be extended to include other diagnostic tests, e.g. pathology results.

### 2.2. Computer-aided analysis techniques

CAD techniques for lung nodules are typically automated systems used as a second reader. This means that a radiologist reviews the CT exams using standard practices looking for nodules. Subsequently, they can activate the automated system, which highlights nodules it has detected. The radiologist then has the option of accepting or rejecting additional CAD findings. Here, CAD serves as a double checker for nodule detection.

**Figure 2:**
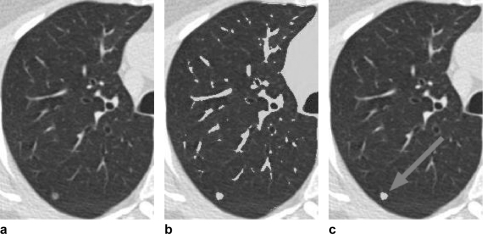
(**a**) Original CT image of the right lung. (**b**) Result of attenuation thresholding in the lung field, with ROIs corresponding to blood vessels and pulmonary nodule in gray. (**c**) Automatically detected nodule (gray with arrow) following classification step.

The details of our CAD system have been described previously, but we provide an outline here. Nodules are typically more radio-dense than the surrounding lung parenchyma, thus having a higher X-ray attenuation (appearing brighter in a CT image against the dark background of the lung [Figure 2a]). Attenuation thresholding and 3D region-growing techniques are applied to create regions-of-interest (ROIs) in the image, which correspond to contiguous sets of voxels that have an X-ray attenuation above a defined threshold. As shown in Figure 2b, this generates many ROIs, mostly from parenchymal blood vessels and airway walls that have a similar attenuation to nodules. The challenge for a detection system is therefore to discriminate nodules from the bronchovascular anatomy—a challenge shared with visual detection.

The CAD system uses pattern classification techniques to discriminate between nodules and vessels. From each ROI the system computes quantitative features that characterize the ROI. Primary features used in this task include ROI volume and shape. For an ROI consisting of a set of voxels, S, the volume and shape features are computed as follows:

$$\begin{array}{l} Volume=\sum_{p\in S}x\_voxel\_size(p)*y\\ \;\;\;\;\_voxel\_size(p)*z\_voxel\_size(p) \end{array}$$

where x_voxel_size(p) is the size of the voxel, p, in the x-direction, with similar definitions in the y- and z-directions.

$$\mathrm{Sphericity}=100*\frac{3\nu }{4\pi r^{3}}$$

where, r = 0.5 * [maximum dimension (x, y or z) of object’s bounding box], and v = volume of the object.

A perfectly spherical object will have a maximum sphericity feature value of 100. The more elongated the object, the lower the sphericity value.

**Figure 3:**
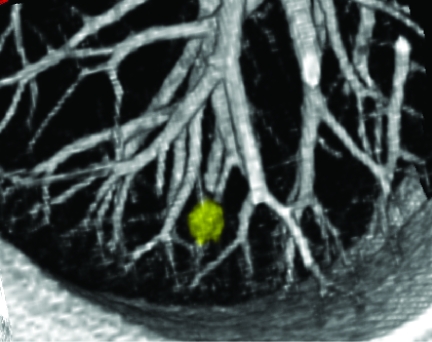
3D rendering of a pulmonary nodule and blood vessels adjacent to the pleural surface.

The pattern classifier contains a model of the expected appearance of nodules and vessels in terms of these features. The details are provided in (4,12), but the model reflects that vessels are long, thin tubular structures in 3D and thus have a low sphericity value, whereas nodules have a higher sphericity value. This is illustrated in Figure 3 showing a 3D rendering of a nodule and adjacent vessels. The system uses fuzzy logic to compute a confidence score indicating whether a given ROI has features consistent with a nodule. ROIs with high confidence for a nodule are presented to the user as shown in Figure 2c. The output of the CAD system is written to a row in the Segmentation_Results table. The row is associated with rows in the Image_Series and Segmentation_ Model tables to indicate the image data set and classification model from which the CAD results were derived.

As part of clinical trials, diameter and/or volume measurements are required to assess nodule size following detection. To perform these measurements, nodule boundaries must be identified on each CT image slice, also known as segmentation. Lung nodules can have irregular and/or poorly defined margins. Therefore, the architecture also includes semi-automated tools for performing a more accurate definition of nodule boundaries than that provided by the detection system. These tools allow a user to apply different attenuation thresholds and interactively edit nodule boundaries (7). The edited segmentation results (ROIs) are stored in the Marking table as well as meta data relating to the radiologist who made the edits (in the Marking_Reviewer table). Following segmentation, diameters and/or volumes are computed (see Equation [Section 2.2]). These measurements are stored in the corresponding table shown in Figure 1.

The CAD architecture also includes capabilities for automatic tracking of a given nodule between serial CT exams. The tracking procedure uses nodule coordinates relative to anatomic landmarks (measured on the baseline scan) to automatically relocalize on subsequent scans (4).

### 2.3. Data mining and reporting

**Figure 4:**
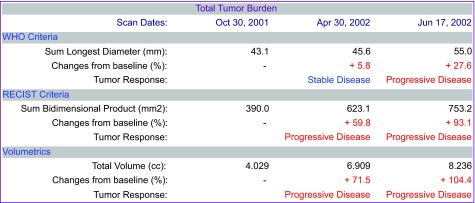
Report from CAD measurement system showing diameter and volume measurements and percentage changes from baseline. From these changes disease stability or progression is determined.

The data model described in Section 2.1 supports queries to retrieve serial nodule volumes from a given subject in a clinical trial. Given a subject ID, the Patient table can be queried to retrieve all CT image series associated with the subject, and then all nodule ROIs associated with the series. From each ROI, the system can compute volume and generate a report of the change in nodule volume between exams. The architecture supports web-based reporting of these results for a given patient. The report includes volumes and diameters, and applies World Health Organization (WHO) and Response Evaluation Criteria in Solid Tumors (RECIST) criteria (see Figure 4). The architecture also supports 3D visualization of nodule changes (Figure 3).

The data model also supports more complex queries over larger populations (e.g. CAD performance testing). To perform an analysis on all patients from a given clinical trial, a query can be performed on the table which associates experiments and image series through the primary keys of their respective tables (as described in Section 2.1). The ROIs from these images then can be retrieved as described above. In addition, a query can be applied to retrieve all nodule ROIs regardless of the associated experiment, effectively pooling subjects across trials. The would allow data mining to determine statistics regarding nodule growth rates (doubling times) based on data in the Measurements table.

### 2.4. CAD evaluation studies

To illustrate the architecture utilization, we describe two experimental trials aimed at evaluating CAD technology.

#### 2.4.1. Computer-aided detection of lung nodules

The purpose of this preliminary study was to evaluate the accuracy of a CAD system in detecting lung nodules on CT exams and to assess its potential as a second reader. The details of the experimental design are provided in (6), but we outline them here.

This study involved 29 subjects from the database who had undergone thin-section CT imaging for known or suspected lung nodules, as part of routine clinical practice. Images were acquired with a slice thickness and reconstruction interval ≤1 mm. For each subject, the scan analyzed in this study had limited longitudinal coverage of around 20 mm, giving 20–40 images per series.

Providing a gold standard for CAD evaluation, three experienced thoracic radiologists visually determined, by consensus review, the presence/location of nodules on the 29 CT scans. Then, 14 of the cases were used in CAD system development (as described in [6]); the remaining 15 cases used in the evaluation studies. The automated CAD system was run on these 15 cases and the sensitivity of nodule detection was measured along with the false positive rate (bronchovascular structures incorrectly marked as nodules). A thoracic radiologist also read the cases under two reading conditions: 1) conventional un-assisted read and 2) after CAD output was made available. The radiologist’s sensitivity for nodules pre- and post-CAD assistance was measured.

#### 2.4.2. Assessment of lung nodule size

The purpose of this study was to investigate the agreement between three techniques for quantifying change in nodule size (7).

The study involved 15 subjects from the database (not those used in the CAD evaluation). The subjects were undergoing therapy for metastatic lung cancer (colorectal, renal cell, or breast primary). Subjects were imaged with a thin-section CT protocol, with slice thickness ≤3 mm, at baseline, and at two follow-up visits of 1–4 month intervals.

From the 15 subjects, a total of 32 lesions were selected for measurement. The three size measurement techniques investigated were:

1. 1D longest diameter measurement made manually with electronic calipers on the axial image where the lesion appears largest;
2. 2D diameter measurements obtained using the longest diameter and the largest perpendicular diameter measured on the same axial image;
3. 3D volume measurement made by semi-automated segmentation of the nodule as described in Section 2.2 and volume computation using Equation (Section 2.2).

Following the WHO and RECIST criteria, the 1D and 2D measurements were used to categorize patients into four treatment response classifications: progressive, stable, partial and complete. Volumetric criteria were used to classify response based on 3D measurements. Response classifications were compared using the three measurement techniques.

## 3. Results

### 3.1. Computer-aided detection of lung nodules

The 15 subjects used to evaluate the CAD system had a total of 79 nodules (mean diameter: 3.34 mm), 57 of which were micronodules <3 mm in diameter.

The CAD system automatically detected 78% of these nodules, with an average of 15 false positives per data set.

The radiologist independently (without CAD assistance) achieved a sensitivity of 62% with 0.3 false positives per data set. When CAD was made available, the radiologist’s sensitivity increased to 80% with no change in false positive rate.

### 3.2. Assessment of lung nodule size

Thirty-two lesions from 15 patients were analyzed. Because each patient had a baseline and two follow-up scans, this yielded 30 response classifications for each measurement technique. The 1D and 3D measurements were concordant in 29 of 30 classifications, while the 2D and 3D measurements were concordant in 23 of 30 classifications. Level of agreement among the three methods was measured using a kappa statistic (K): 1D compared with 3D, K = 0.739 ± 0.345 (visits 1, 2) and 0.273 ± 0.323 (visits 2, 3); 2D compared with 3D, K = 0.655 ± 0.325 (visits 1, 2) and 0.200 ± 0.208 (visits 2, 3). Agreement among the methods for round and ovoid nodules was also fair to poor.

### 3.3. Overall database size

The database currently stores data from 12 clinical trials and contains images received from a total of 81 sites. The total number of patients in the database is 3,017; the number of CT series is 24,959 and the number of axial images is 3,051,693.

The majority of subjects are from clinical trials relating to lung cancer screening and diagnosis, and other smoking related-diseases (particularly emphysema). The largest trials and data mining queries have been in the area of emphysema treatment (13); however, we expect to make similar large data mining queries for lung cancer.

## 4. Discussion

CAD and measurement systems have great potential to assist in nodule assessment as part of clinical trials. Other studies have since shown similar trends in terms of improving reader performance in nodule detection (2,14). In fact, these tools may be essential for the analysis of high-resolution CT images to be feasible on large cohorts of subjects. Manual segmentation and volume measurement of nodules would likely be too time-consuming for radiologists. CAD tools could enable large amounts of data to be extracted for mining.

CAD techniques are a subject of extensive research, and though sensitivities for nodules are improving, false positive rates remain unacceptably high. The challenge in algorithm development is to reduce false positives while maintaining high sensitivity.

Although 1D and 2D tumor size measurement techniques are standard practice in clinical trials, the CAD tools described in this paper may enable volumetric measures to be made routinely. As described in Section 3.3, and in more detail in (7), preliminary results suggest that response rates based on volume may yield significantly different outcomes to those from 1D and 2D methods. Volume may provide a more reproducible measure of nodule size, particularly for irregular lesions, but, if volume is to become the gold standard for tumor size measurement, then volumetric response criteria will need to be defined.

The experiments presented demonstrate the ability to organize and mine CAD results within this architecture. In this case, mining is to evaluate the CAD technology itself; however, the mining could also involve more complex queries to advance our understanding of phenotypic signatures of lung cancer or for therapeutic clinical trials. Though to date our lung cancer experiments have used only small numbers of cases from the database, emphysema studies have used much larger numbers (13), and the infrastructure can handle it. Future lung cancer related experiments will use data from much larger cohorts.

The infrastructure currently incorporates QIWS and a research CAD system developed at UCLA. However, the data model can readily accommodate another CAD system and another DICOM-compliant workstation with ROI display and editing capabilities. New components would require a data mediation layer to allow access to the database. For an alternative CAD system to be “plugged-in” it would need to be runnable as a stand-alone executable (i.e. not be tied to its own viewing workstation) so that it could be called from QIWS or another viewing workstation.

## 5. Conclusion

In this paper we have described a CAD system within an architecture to store and organize image and related data from large cohorts of subjects, acquired at multiple institutions. We have reported on CAD tools for extracting and storing lung nodule measurements and demonstrated their capabilities in two experimental studies.

CAD tools enable efficient and reproducible analysis of large numbers of images. The architecture has great potential for large cancer clinical trials involving imaging.
